# Exploring Agency in Dementia: Care Staff Perspectives and Recommendations

**DOI:** 10.1002/alz.086504

**Published:** 2025-01-09

**Authors:** Annikka Immonen

**Affiliations:** ^1^ University of Turku, Turku, Varsinais‐Suomi Finland

## Abstract

**Background:**

My dissertation focused on the cultural and historical images of agency in person with dementia. In this partial study I examined care staff, who were also in a caregiver role, and their perceptions of how neurocognitive disorders (NCDs) change agency of a person. This role enabled for them to observe the entire disease process.

**Methods:**

The research utilized data from a survey of care staff (n = 191) and Finnish dementia care textbooks spanning 1985 to 2019 (77 books). The theoretical framework consisted of communicative (Holzkamp), dynamic (Thomas and Znaniecki, Jyrkämä), and embodied (Kontos) agency of a person with dementia.

**Results:**

Before the onset of illness, care staff depicted their loved ones as social, cheerful, and purposefully active individuals with various possibilities for activities and skills to lead unique lives. The narratives of agency emphasized purposeful and intentional activity, as well as physical engagement.

As neurocognitive disorders progressed, care staff observed a decline in agency, particularly linked to cognitive impairment. The narratives highlighted loss of abilities, memory, and social agency. Embodied agency was underemphasized in the narratives. The non‐verbal body language of persons with dementia was weakly presented. Agency was interpreted through the lens of spoken language and cognitive functioning.

**Conclusion:**

In both textbooks and accounts from care staff, the non‐verbal body language of people with dementia was inadequately portrayed.

To optimally fulfil the agency of people with dementia, the study recommends actively observing their embodied agency and assessing their needs based on these observations. In response to the research findings, a remote learning course is currently under development to assist care staff in recognizing and supporting embodied agency of people with dementia.

